# The Other Cheek

**Published:** 1892-07-02

**Authors:** 


### The Doctor's Armchair.

### THE OTHER CHEEK.

One of the most important revolutions that science is destined to effect in the habits of civilised people is that of teaching them the great advantage of conducting their daily business without passion. *Ira furor brevis est*, says the Latin school-book of our early days. Rage, at any rate, if it be sufficiently passionate and uncontrolled, is madness; and, unhappily, we cannot always qualify it by the word "brief." The following case, if regarded steadily for some time from the scientific and philosophic standpoints, will afford a kind of mental "cud" which may be ruminated with both intellectual and moral profit.

There has apparently been a sharp quarrel between the Rev. Mr. Blackmore, rector of the parish and lord of the manor of Morchard-Bishop, and Mr. William Francis Tronson, M.R.C.S., the one doctor of the same parish. Of the merits of the quarrel we know nothing. But the magistrates at Crediton appear to have considered that the clergyman was in the wrong, and accordingly they fined him forty shillings for assaulting the doctor. If the matter had ended here it would have been of comparatively little interest to the general public, for there seems to be no more reason in the abstract why a clergyman and a doctor should not settle their quarrels by the primitive method of fisticuffs than why a French editor and a soldier should not settle theirs by the almost equally elementary methods of toy pistols or Lilliputian swords. Our chief objection is that these methods have the double disadvantage of looking extremely silly, and of providing no basis of settlement which can properly be called "equitable."

It is the after conduct of the Rev. Mr. Blackmore, rector of the parish and lord of the manor, which strikes us who live in this scientific epoch as being so grievously unscientific. *The British Medical Journal* informs us that after hearing the magistrate's decision, the rector wrote to the doctor in the following terms: "When the court's proceedings are over, I have firmly decided to act as follows: As I am now likely to remain at Morchard through the breakdown in the negotiations for my leaving, I shall rigidly boycott you with all my tenants; and if any of them venture to employ you professionally after receiving my circular, they will immediately have notice to quit, whoever they may be. As I have eighty houses in my hands, either as rector or lord of the manor, the effect upon your position will be pretty considerable. I shall then advertise in the medical papers, or through the agents, that there is a good opening here for a doctor to begin practice without purchase, and I shall offer board and residence in the rectory free to any gentleman who will come and establish himself. I have only to do this to take your practice completely away, with the exception of a few personal friends you may have. Lots of the parishioners have told me they will gladly welcome a new man. This is my answer to your challenge, and you ought to know by the many defeats the farmers have met with at my hands that when I make up my mind I leave no stone unturned to gain my end."

Now this gentleman has a business—the business of a teacher. His calling is not that of an ordinary tradesman or farmer, which can be taken up or laid down by any person who so chooses at any moment. But it is a business to which he has been set apart by ecclesiastical and State authorities, and by a series of solemnities of the most impressive kind. A chief part of this business is the teaching of all the people of his parish this one admittedly peculiar but very definite principle: "If a man smite thee on the one cheek, turn to him the other also." Now we do not here pronounce whether this principle is philosophical or unphilosophical, or whether in practice it is possible or impossible. What we do say is that this gentleman has vowed in the most solemn manner, and under the most solemn circumstances, to believe it, to teach it, and to practice it. The way in which he actually does practice it may be seen by a reference to his letter addressed to Dr. Tronson, which we have just quoted.

The object we have in view in giving prominence to the incident is not the defence of Dr. Tronson, or the delivery of a homily to the Rev. Mr. Blackmore. It is the consideration of the question whether or not a man is under any absolute obligations to practice in private what he professes in public, and what he usually lives by. And this purpose we do not wish to carry out with particular reference to the clergy, but rather with special reference to the members of our own profession. The reason why the incident has been selected as a text is the glaring absurdity which it so well displays of an educated man making a profession of one set of principles and practising an exactly opposite set.

It is a well-known fact that a certain number of medical men practice physic, live by it, and yet do not hesitate on occasions to publish the circumstance that they have little or no faith in it. Those men are not always the uncultured and the mentally incapable. On the contrary, some of them are of more than average education and intellectual capacity. But because they have no particular aptitude for the art of healing, as distinguished from the science that busies itself about medicine, therefore they experience a frequent want of success in treatment; and therefore also they fail to find pleasure in their work, and in course of time they come, in the most natural way, to lose all faith in the principles of their art.

When a man has reached this stage in medicine, or any other profession, he occupies a position, if he still continues to practice his art, which is at once both absolutely ridiculous and absolutely immoral. It requires no unusual clearness of intellect to see the more than childish folly and inconsequence of the Rev. Mr. Blackmore's behaviour when he wrote his extraordinary letter to Dr. Tronson. One feels that the poor man was out of his mind; beside himself with the most impotent and ludicrous rage. He not only tears his passion to tatters in his letter, but cuts it up into the smallest and most infinitesimal pieces, and makes every separate piece so altogether futile that it would not bring down even a midge on the wing. The man's folly is so stark and complete that it would be impossible for any effort to go beyond it. And yet this gentleman is not one whit more foolish or one whit more wicked than is a doctor who practises the profession of medicine whilst he openly or covertly avows his disbelief in it. The amusing, but, happily, unparalleled, case of Mr. Blackmore offers one of the most striking contrasts between profession and practice which it has ever been our lot to meet with. Every man who is intellectually honest may give himself a wholesome moral shock by the study of it.

